# Concomitant pulmonary embolism and myocardial infarction due to paradoxical embolism across a patent foramen ovale: a case report

**DOI:** 10.1093/ehjcr/ytx010

**Published:** 2017-11-23

**Authors:** Mohammad Alkhalil, Thomas J Cahill, Henry Boardman, Robin P Choudhury

**Affiliations:** 1 Division of Cardiovascular Medicine, Radcliffe Department of Medicine, University of Oxford, John Radcliffe Hospital, Headley Way, OX3 9DU Oxford, UK; 2 Acute Vascular Imaging Centre, Radcliffe Department of Medicine, University of Oxford, John Radcliffe Hospital, Headley Way, OX3 9DU Oxford, UK; 3 Department of Cardiology, Oxford Heart Centre, University of Oxford, John Radcliffe Hospital, Headley Way, OX3 9DU Oxford, UK

**Keywords:** ST-segment elevation myocardial infarction, Pulmonary embolism, Thrombectomy, Thrombolysis, Patent foramen ovale

## Abstract

Concomitant acute myocardial infarction (MI) and pulmonary embolism (PE) is exceedingly rare. However, establishing the diagnosis early is essential, since delay in treating the patient may lead to a potential fatal outcome. Right ventricular (RV) infarction in the setting of inferior ST-segment elevation MI (STEMI), coupled with acute massive PE confers particular risk due acute RV failure and low cardiac output, threatening survival. We report a rare case of concomitant PE and inferior STEMI in a 43-year-old woman with a history of acute chest pain. She was haemodynamically compromised, with Type I respiratory failure but lack of signs of heart failure. Early recognition of dual pathologies prompted administration of thrombolytic therapy and simultaneous right coronary artery thrombectomy to treat PE and STEMI. Prompt clinical diagnosis and delivery of targeted therapies adapted for the specific clinical presentation may have averted fatal outcome.

Learning points

- Concomitant acute myocardial infarction and pulmonary embolism is rare but a potentially fatal combination. Early recognition of this clinical entity can facilitate delivery of targeted therapy, adapted for the specific clinical presentation, and may avert a fatal outcome.
- Type I respiratory failure in the context of acute myocardial infarction and lack of heart failure signs should prompt thinking of second pathology like pulmonary embolism.

## Introduction

Concomitant acute myocardial infarction (MI) and pulmonary embolism (PE) is rare, but establishing this dual diagnosis early is essential, since delay in appropriate therapy may lead to a fatal outcome. Right ventricular (RV) infarction in the setting of inferior ST-segment elevation MI (STEMI) coupled with acute massive PE confers a particular risk due to acute RV failure and cardiogenic shock.^1^ We report a rare case of concomitant PE and inferior STEMI, where early recognition prompted simultaneous right coronary artery (RCA) thrombectomy and administration of thrombolytic therapy to treat pulmonary embolism.

## Timeline

**Table:** 

Time	Events
Initial presentation (Day 1)	Onset of chest pain and initial assessment revealing inferior ST-segment elevation myocardial infarction.
	Transfer to cardiac catheter laboratory where coronary angiography took place via the right radial artery.
	Administration of tissue plasminogen activator (tPA) via the intracoronary route, followed by systemic dose intravenously. Subsequently, multiple aspirations using an Export thrombectomy catheter was performed.
	Transfer to radiology department for computed tomography pulmonary angiogram (CTPA), which confirmed acute pulmonary embolism.
Day 2	Transthoracic echocardiogram with bubble-agitated saline revealed a patent foramen ovale (PFO).
2 months	Right heart catheterization showing mean pulmonary arterial pressure of 33, wedge pressure of 11, cardiac output of 4.7 L/min (index 2.35) with pulmonary vascular resistance of 5.3 Wood units. The patient managed 80 m on 6-min walk test.
Follow-up (6 months)	Pulmonary endarterectomy with subsequent successful PFO closure.
Outpatient clinic (12 months)	Marked improvement in patient’s symptomatic status, including significant improvements in 6-min walking test (411 m).

### Patient information

A 43-year-old woman presented with a history of 2 h of central chest pain associated with breathlessness. She had no cardiac risk factors. Her medical history was significant for PE (diagnosed 5 years ago), managed with apixaban, but with poor adherence.

### Physical examination

On arrival, the patient was profoundly hypotensive with a blood pressure of 60/30 mmHg. She was tachycardiac with a heart rate 115 b.p.m. and peripherally shut down. Cardiovascular examination revealed a parasternal heave, but no murmurs were audible. The lung fields were clear. There was no clinical evidence of deep vein thrombosis.

### Diagnostic assessment

A 12-lead electrocardiogram (ECG) showed inferior ST-segment elevation in leads II, III, and aVF, in keeping with acute MI. An arterial blood gas (on room air) demonstrated acute Type I respiratory failure with an arterial partial pressure of oxygen of 5.5 kPa.

### Interventions

**Figure 1:**
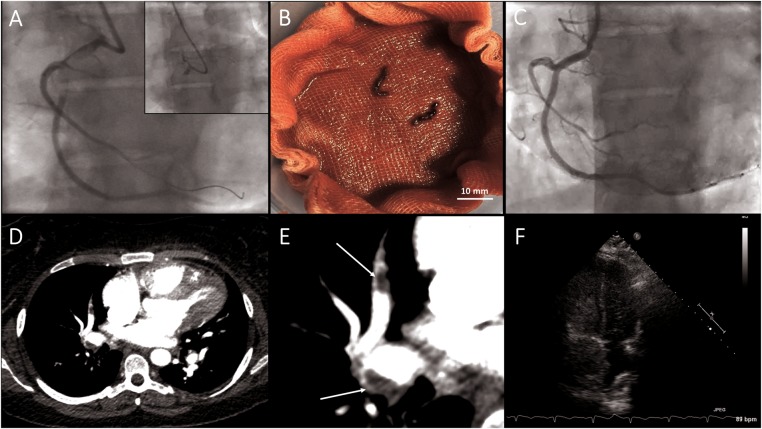
Paradoxical embolism leading to occluded right coronary artery (successfully aspirated) and extensive pulmonary embolism.

The patient was transferred urgently to the Cardiac Catheter Laboratory. Coronary angiography via the right radial artery showed complete occlusion of the RCA, with a large thrombus straddling a bifurcation in the proximal artery (RCA). The left coronary system was normal. A guidewire was passed across the occlusion, and the origin of the right ventricular branch was identified (*Figure 1A*). Given the history of PE and poor compliance with anticoagulant therapy, acute Type I respiratory failure with clear lung fields, a concurrent diagnosis of acute PE was suspected. Empirical intracoronary tissue plasminogen activator (tPA; alteplase, 15 mg) was given via the intracoronary route. Multiple aspirations (including of the RV marginal branch) using an Export thrombectomy catheter (Medtronic Vascular, Minneapolis, MN, USA) obtained three large clots (*Figure 1B*). This established thrombolysis in myocardial infarction (TIMI) III flow downstream in the RCA. The lack of angiographic evidence of atherosclerotic plaque suggested a possible thrombo-embolic infarct. Concurrently, the blood pressure started to improve reaching the systolic pressure of 100 mmHg but with residually poor oxyhaemoglobin saturation of 75%.

After stabilization, the patient was transferred directly from the Cardiac Catheter Lab for urgent computed tomography pulmonary angiogram. This confirmed acute PE with extensive clot burden in the proximal right pulmonary artery (*Figure 1D* and *E*). The full tPA protocol was completed intravenously. A subsequent transthoracic echocardiogram with bubble-agitated saline revealed a patent foramen ovale (PFO) (*Figure 1F*), with preserved left ventricular function, but a dilated right ventricle with elevated pulmonary artery pressure. The patient was electively scheduled for right heart catheterization, which demonstrated mean pulmonary arterial pressure of 33 mmHg, wedge pressure of 11 mmHg, cardiac output of 4.7 L/min (index 2.35) with pulmonary vascular resistance of 5.3 Wood units.

### Follow-up and outcomes

The patient underwent surgery for pulmonary endarterectomy (leading to reduction in her pulmonary pressure from 47 mmHg to 18 mmHg) and PFO closure 6 months after the index presentation and was symptomatically well on 12 months of follow-up. Her 6-min walk test was significantly improved from 80 m to 411 m.

## Discussion

Coronary embolism is a recognized cause of acute MI; however, paradoxical embolism is relatively rare.^2^ In an autopsy series of more than 1000 MI patients, coronary embolism accounted for only 5% of the events, with no cases due to paradoxical embolism.^2^ The high prevalence of PFO in echocardiographic studies makes definitive diagnosis of paradoxical embolism challenging.^3^ Rather, three criteria have been proposed to make a presumptive diagnosis: (i) embolisation that was not sourced from left heart but (ii) had originated from the venous system and was (iii) associated with abnormal communication between the venous and arterial circulations, in the form of atrial septal defect or PFO.^4^ The combination of paradoxical embolism with concomitant PE is extremely rare.

Acute PE is frequently implicated as detrimental driver for embolism to travel across a PFO.^5^ This may be explained by the pathophysiological changes that occur following PE. A large PE results in elevated right atrial pressures and systemic hypotension, promoting reversed (right-to-left) blood flow across the PFO.^5^ In a study of 139 patients with a large PE, the presence of a PFO significantly increased the risk of stroke, peripheral embolism, and mortality.^6^

The similarities in clinical presentations between acute PE and MI make a joint diagnosis very challenging, and more often symptoms, and even ECG changes, are attributed to one pathology.^7^ Profound hypotension in the context of acute MI is usually due to cardiac pump failure or mechanical complications such as mitral regurgitation, ventricular septal defect, or wall rupture.^8^ However, in our case, the diagnosis of PE was prompted by extreme hypotension combined with profound hypoxaemia, without pulmonary oedema and out of keeping with an acute inferior MI. The combination of inferior/RV infarctions and pulmonary arterial obstruction with increased pulmonary vascular resistance was considered particularly adverse. The haemodynamic benefits are greatest when thrombolysis was initiated as early as possible, reinforcing the importance of the immediate recognition of this problem and delivering appropriate treatment.^9^^,^^10^

The use of thrombus aspiration has been proposed as adjunctive treatment to restore coronary perfusion in the context of STEMI.^11^ In the latest European Society of Cardiology (ESC) guidelines, the *routine* use of thrombectomy catheter is no longer recommended; however, it has to be targeted towards specific group of patients. In a recent meta-analysis, patients with a large thrombus burden undergoing thrombectomy demonstrated mortality benefits.^12^ Whether intracoronary lytic therapy may facilitate successful thrombus aspiration is currently being studied in the T-Time trial.^13^

During the acute setting, closure of PFO is controversial and should be individualized. Although theoretically it may help prevent further recurrence of paradoxical emboli, the PFO may serve to offset the elevated right pressure and maintain cardiac output at the expense of low systemic saturation.^14^^,^^15^ In fact, some reports recommend against PFO closure in the setting of right elevated pressure.^14^ However, in our case, a successful pulmonary endarterectomy leading to reduction in pulmonary pressure has rationalized the decision to close PFO.

In conclusion, we reported a rare case of concurrent acute MI caused by paradoxical emboli, associated with, and likely triggered by acute PE. In this case, prompt clinical diagnosis and simultaneous delivery of targeted therapies adapted for the specific clinical presentation have led to an excellent long-term outcome.

## Funding

NIHR Oxford Biomedical Research Centre (R.P.C. and M.A.).

**Consent:** The author/s confirm that written consent for submission and publication of this case report including image(s) and associated text has been obtained from the patient in line with COPE guidance.

**Conflict of interest:** none declared.
